# Features of *KRAS-*mutated patients with chronic myelomonocytic leukemia with and without blast transformation in a national (ABCMML) and international cohort (BIOPORTAL)

**DOI:** 10.1007/s10354-025-01099-3

**Published:** 2025-07-22

**Authors:** Melanie Weissenbacher, Klaus Geissler

**Affiliations:** Medical School, Sigmund Freud University, Sigmund Freud Platz 3, 1020 Vienna, Austria

**Keywords:** Chronic myelomonocytic leukemia, *KRAS*, Austrian biodatabase for chronic myelomonocytic leukemia, BIOPORTAL, Acute myeloid leukemia, Chronische myelomonozytäre Leukämie, *KRAS*, „Austrian Biodatabase for chronic myelomonocytic leukemia“, BIOPORTAL, Akute myeloische Leukämie

## Abstract

Big data collected in large international cooperations nowadays allow validation of findings from traditional national patient cohorts for proving consistency. In this study we compared findings in *KRAS*-mutated patients of the Austrian biodatabase for chronic myelomonocytic leukemia (ABCMML) with that from the CMML cohort documented in cBioPortal. It was consistently shown in both CMML cohorts that *KRAS* mutations were not associated with shorter overall and acute myeloid leukemia (AML)-free survival. In both cohorts, phenotypic features such as leukocytes, hemoglobin, and circulating blasts were not significantly different between patients with and without *KRAS* mutations. However, the proportion of patients with thrombocytopenia was higher in *KRAS*-mutated patients in the BIOPORTAL cohort but not in the ABCMML cohort. The percentage of *KRAS* mutations significantly increased in blast transformation (from 9.8 to 21.7%), as shown in the ABCMML cohort. These data may suggest a pathogenetic role of *KRAS *mutations in CMML-associated AML.

**Supplementary Information:**

The online version of this article (10.1007/s10354-025-01099-3) contains supplementary material, which is available to authorized users.

## Abstract (de)

Große Datenmengen aus internationalen Kooperationen ermöglichen die Validierung nationaler Patientenkohorten zur Überprüfung der Konsistenz von Daten. In dieser Studie verglichen wir die Ergebnisse von Patienten mit *KRAS-*Mutation der nationalen „Austrian Biodatabase for chronic myelomonocytic leukemia“ (ABCMML) mit Patienten der internationalen Plattform cBioPortal. Es kann in beiden Kohorten mit chronischer myelomonozytärer Leukämie (CMML) übereinstimmend gezeigt werden, dass *KRAS*-Mutationen nicht mit einem schlechteren Gesamtüberleben und von akuter myeloischer Leukämie (AML) freiem Überleben assoziiert sind. Auch phänotypische Merkmale wie Leukozytenzahl, Hämoglobin und zirkulierende Blasten waren in beiden Kohorten nicht unterschiedlich. Der Anteil von Patienten mit Thrombozytopenie bei Patienten mit *KRAS*-Mutation war in der BIOPORTAL-Kohorte, allerdings nicht in der ABCMML-Kohorte erhöht. Der Prozentsatz von *KRAS*-Mutationen stieg, wie in der ABCMML-Kohorte gezeigt werden konnte, signifikant in der Blastentransformation an (von 9,8 auf 21,7 %). Diese Daten lassen eine pathogenetische Rolle der *KRAS*-Mutation bei CMML-assoziierter AML vermuten.

## Introduction

Chronic myelomonocytic leukemia (CMML) is a rare, genotypically and phenotypically heterogenous hematologic malignancy of elderly people with an intrinsic risk of progression and transformation into secondary acute myeloid leukemia (AML). With regard to the presence of myeloproliferation, CMML was originally subdivided into myeloproliferative disorder (MP-CMML; white blood cell count (WBC) > 13 × 10^9^/L) and myelodysplastic syndrome (MD-CMML; WBC ≤ 13 × 10^9^/L) by the FAB criteria [1, 2]. Since CMML is characterized by features of both MDS and MPN, the World Health Organization (WHO) classification of 2002 assigned CMML to the mixed category, MDS/MPN [3]. After the 2016 revision of the WHO classification of myeloid neoplasms and acute leukemia [4], updated diagnostic criteria for CMML were recently reported by two groups [5, 6]. CMML patients can have highly variable outcomes, suggesting that several factors determine the course of disease and the causes of death in these patients [7–13].

Recently, we reported the Austrian biodatabase for CMML (ABCMML). In the ABCMML, epidemiologic, hematologic, biochemical, clinical, immunophenotypic, cytogenetic, molecular, and biologic data of patients with CMML have been collected from different Austrian centers for 40 years [14]. It has been shown to be a representative and useful real-life data source for biomedical research.

Due to the molecular heterogeneity of CMML, it is important to know the meaning of molecular characteristics in order to be able to offer the patient the best possible management in their individual situation. Some studies have analyzed the impact of molecular aberrations on the clinical outcome and phenotype of disease, but the findings of most studies were not validated in independent cohorts. However, a prognostic parameter should not enter clinical practice unless it has been demonstrated that it performs a useful role. External validation denotes evaluation of the performance of a prognostic parameter in a sample independent of that used to develop the model [15].

Big data containing a huge number of datasets from international large consortium efforts are now available in many cancer entities including CMML. The cBioPortal platform is such a collection of big data that aims to build a platform to support clinical decisions for personalized cancer treatment [16]. Moreover, due to the large number of well-characterized patients, it is a perfect source of data for validation of findings in traditional, sometimes much smaller patient cohorts. In this study, we used data from CMML patients documented in cBioPortal to validate the features of *KRAS*-mutated CMML patients who have been analyzed in the ABCMML.

## Patients and methods

### Patients

#### ABCMML analysis

Recently, we have shown that the ABCMML may be used as a representative and useful real-life data source for biomedical research [14]. In this database, we retrospectively collected epidemiologic, hematologic, biochemical, clinical, immunophenotypic, cytogenetic, molecular, and biologic data of patients with CMML from different centers. The diagnosis of CMML and leukemic transformation were according to the WHO criteria [2–4]. Clinical and laboratory routine parameters were obtained from patient records. A detailed central manual retrospective chart review was carried out to ensure data quality before analysis of data from institutions. In the ABCMML cohort, CMML patients without transformation and patients with CMML-associated acute myeloid leukemia (AML) were included. In 327 CMML patients without transformation, mutation data were available to analyze overall survival (OS), AML-free survival, and differences in phenotypic parameters between mutated and wildtype patients. A total of 46 patients with CMML-associated AML and molecular characteristics could be analyzed regarding overall survival and phenotypic characteristics. This research was approved by the ethics committee of the City of Vienna on 10 June 2015 (ethic code: 15-059-VK).

#### cBioPortal analysis

The cBioPortal for cancer genomics provides visualization, analysis, and download of large-scale cancer genomics datasets [16]. We selected the myelodysplastic syndromes dataset containing 399 CMML cases with data including age, sex, white blood cell count (WBC), hemoglobin (Hb), platelets, overall survival, AML-free survival, bone marrow (BM) blasts, circulating blasts, cytogenetics, and gene mutations (http://www.cbioportal.org) to analyze OS, AML-free survival, and differences in phenotypic parameters between mutated and nonmutated patients. Data from patients with CMML-associated AML, however, are not shown in this database.

### Statistical analysis

The log-rank test was used to determine whether individual parameters were associated with OS and AML-free survival. Overall survival was defined as the time from sampling to death (uncensored) or last follow-up (censored), and AML-free survival was defined as the time from sampling to transformation into AML or death (uncensored) or last follow-up (censored). Dichotomous variables were compared between different groups using the chi-square test. The Mann–Whitney U test was used to compare two unmatched groups when continuous variables were not normally distributed. Results were considered significant at *p* < 0.05. Statistical analyses were performed with SPSS v. 27 (IBM Corp., Armonk, NY, USA); the reported *p*-values were two sided. In the ABCMML database, mutations with a variant allele frequency (VAF) of at least 5% and in the cBioPortal platform with an VAF of at least 2% are considered positive.

## Results

### Characteristics and percentages of *KRAS* mutations in CMML patients without blast transformation

The baseline characteristics of both CMML cohorts are shown in supplementary table 1. Overall, 327 patients in the ABCMML cohort and 399 patients in the cBioPortal cohort were analyzed. As seen in other CMML series, there was a male predominance among CMML patients in both cohorts and more than half of the patients were 70 years or older [14]. All characteristics except leukocytes were comparable between cohorts. The proportion of patients with leukocytes > 13 G/L was significantly higher in the ABCMML cohort as compared to the cBioPortal cohort (57% vs. 32%, *p* < 0.001). The median leukocyte counts were 14.1 vs. 9.2 G/L in these cohorts, respectively. Regarding clinical outcome, median survival was 29.0 months in the ABCMML cohort as compared to 31.6 months in the cBioPortal cohort. The percentages of *KRAS* mutations were 9.7% (32/327) in the ABCMML group and 14.0% (56/399) in the BIOPOPRTAL group.

### Impact of *KRAS* mutations on survival and AML-free survival in CMML patients without blast transformation

**Fig. 1:**
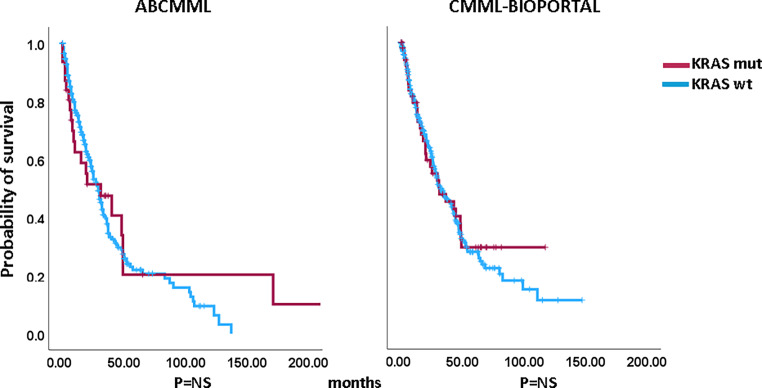
Kaplan–Meier plots for overall survival in CMML patients with and without *KRAS* mutations

**Fig. 2:**
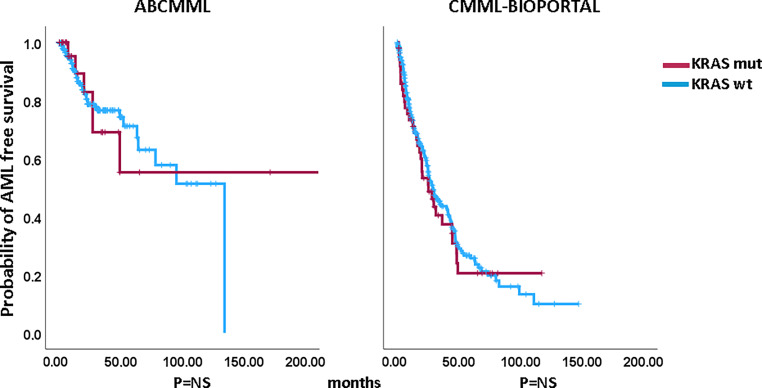
Kaplan–Meier plots for AML-free survival in CMML patients with and without *KRAS* mutations

Figures 1 and 2 show the Kaplan–Meier curves of OS in *KRAS*-mutated (variants and variant allele frequencies are shown in supplementary table 2) and *KRAS*-nonmutated patients in both cohorts. In neither cohort did *KRAS*-mutated patients have significantly inferior survival. The median survival of *KRAS*-mutated patients was 31.0 vs. 29.0 months (*p* = 0.606) in the ABCMML patients and 31.2 vs. 31.7 (*p* = 0.696) months in the cBioPortal patients. Regarding AML-free survival there was also no significant difference between *KRAS*-mutated and *KRAS*-nonmutated patients in either cohort. The median AML-free survival was not reached vs. 134.0 (*p* = 0.992) in the ABCMML cohort and 25.2 vs. 28.6 (*p* = 0.660) months, respectively, in the cBioPortal cohort.

### Laboratory features in the presence or absence of *KRAS* mutations in CMML patients without blast transformation

**Fig. 3:**
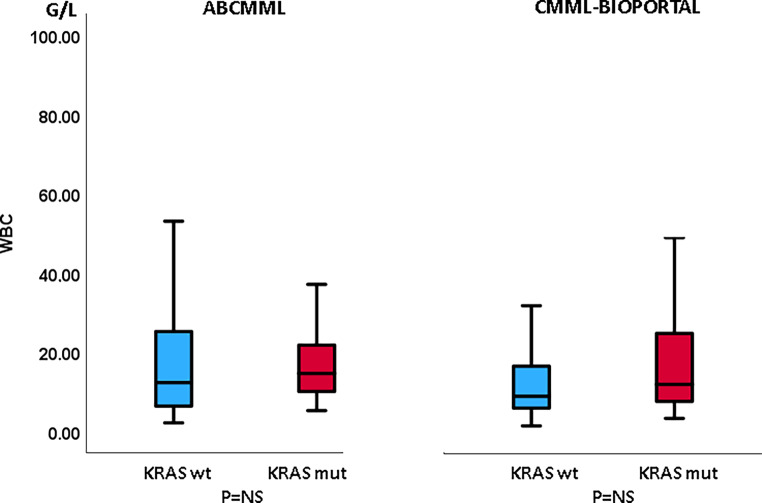
Boxplots showing the distribution of leukocytes in *KRAS*-nonmutated and *KRAS*-mutated CMML patients including median values, minimum values, maximum values, and upper and lower quartiles in both study cohorts

**Fig. 4:**
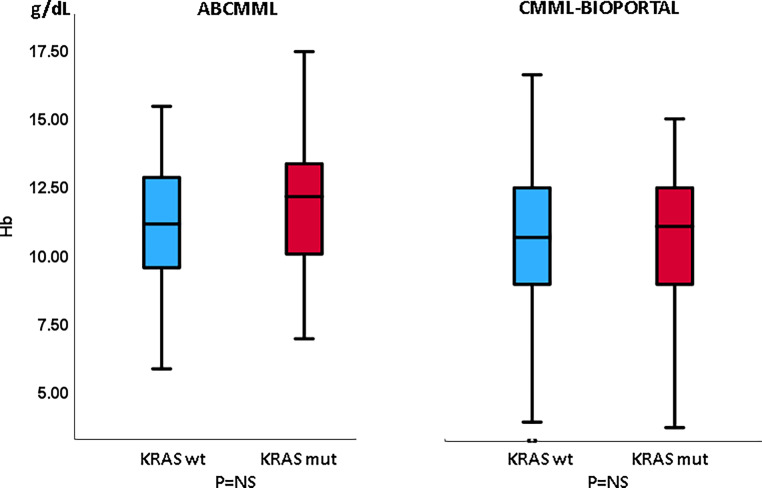
Boxplots showing the distribution of hemoglobin values in *KRAS*-nonmutated and *KRAS*-mutated CMML patients including median values, minimum values, maximum values, and upper and lower quartiles in both study cohorts

**Fig. 5:**
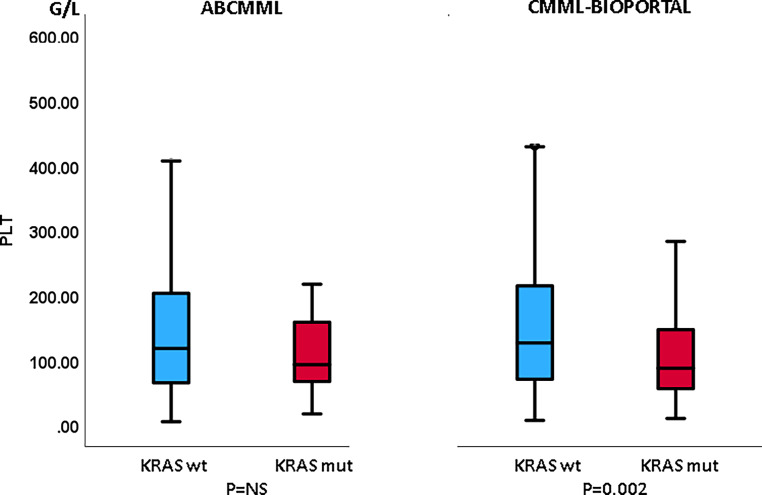
Boxplots showing the distribution of platelets in *KRAS*-nonmutated and *KRAS*-mutated CMML patients including median values, minimum values, maximum values, and upper and lower quartiles in both study cohorts

Table 1 shows the phenotypic parameters in the ABCMML and the cBioPortal patients without transformation. In neither cohort was there a difference regarding leukocytes, hemoglobin, or circulating blasts between *KRAS*-mutated and nonmutated patients. In the cBioPortal cohort the proportion of patients with thrombocytopenia < 100 G/L was higher in patients with a *KRAS* mutation as compared to patients without a mutation. In Figs. 3, 4, and 5 metric values are visualized by boxplots. In the ABCMML cohort, the median values of *KRAS*-mutated and nonmutated patients were 15.0 vs. 12.2 G/L for WBC, 11.1 vs. 12.1 g/dL for Hb, and 101 vs. 118 G/L for platelets, respectively. In the cBioPortal cohort, the median values of *KRAS*-mutated and nonmutated patients were 12.0 vs. 9.0 G/L for WBC, 11.1 vs. 10.7 g/dL for Hb, and 88 vs. 127 G/L for platelets, respectively. As shown in Fig. 5, the metric values for platelets were significantly lower in *KRAS*-mutated patients as compared to nonmutated patients in the cBioPortal cohort but not in the ABCMML cohort.

**Table 1:** Phenotypic features of ABCMML and cBioPortal patients without transformation, including leukocytosis, anemia, thrombocytopenia, and circulating blasts, stratified by the presence or absence of a *KRAS* mutation

Parameter	With *KRAS* mutation	Without *KRAS* mutation	*P*-value
*ABCMML*	(*n* = 32)	(*n* = 285)	–
WBC ≥ 13 G/L	18/31 (58%)	133/284 (47%)	0.260
Hb < 10 g/dL	8/31 (26%)	91/284 (32%)	0.546
PLT < 100 G/L	16/31 (52%)	120/285 (42%)	0.309
PB blasts present	7/24 (29%)	53/240 (22%)	0.446
*cBioPortal*	(*n* = 56)	(*n* = 343)	–
WBC ≥ 13 G/L	23/54 (43%)	98/329 (30%)	0.081
Hb < 10 g/dL	18/56 (29%)	131/340 (39%)	0.360
PLT < 100 G/L	33/56 (59%)	122/333 (37%)	0.002
PB blasts present	12/50 (24%)	76/283 (27%)	0.731

### Survival and laboratory features in the presence or absence of *KRAS* mutations in CMML patients with blast transformation

**Fig. 6:**
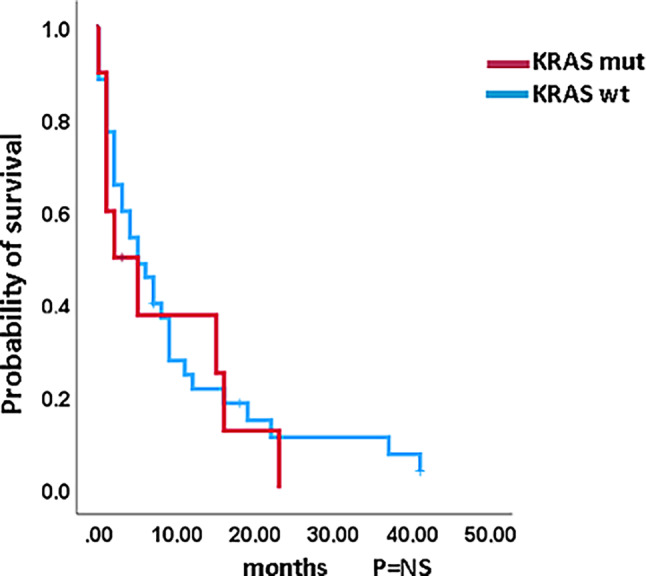
Kaplan–Meier plots for overall survival in patients with CMML-associated AML with and without *KRAS* mutations

The characteristics of patients with CMML-associated AML are shown in supplementary table 3. Information regarding this patient group could only be derived from the ABCMML database, since patients with this diagnosis are not captured in the cBioPortal database. In patients with CMML with blast transformation, the percentage of *KRAS* mutations was 21.7% (10/46). This was significantly higher as compared to CMML patients without transformation (9.8%; 32/327; *p* = 0.016). Figure 6 shows the Kaplan–Meier curves of OS in *KRAS*-mutated (variants and variant allele frequencies are shown in supplementary table 4) and *KRAS*-nonmutated patients with CMML-associated AML. The median OS was 5 months in patients without a *KRAS* mutation and only 2 months in patients with a *KRAS* mutation, although this difference was not statistically different (*p* = 0.655). Table 2 shows the phenotypic parameters in these patients. There was no difference regarding leukocytes, hemoglobin, platelets, and circulating blasts between *KRAS*-mutated and nonmutated patients.

**Table 2:** Phenotypic features of patients with CMML-associated AML from the ABCMML database, including leukocytosis, anemia, thrombocytopenia, and circulating blasts, stratified by the presence or absence of *KRAS* mutations

Parameter	With *KRAS* mutation (*n* = 10)	Without *KRAS* mutation (*n* = 36)	*P*-value
WBC ≥ 13 G/L	8/8 (100%)	26/34 (76%)	0.316
Hb < 10 g/dL	6/8 (75%)	17/34 (50%)	0.258
PLT < 100 G/L	7/8 (88%)	25/34 (74%)	0.655
PB blasts present	6/8 (75%)	23/32 (72%)	1.000

## Discussion

In this study we analyzed a national CMML cohort from Austria (ABCMML) and an international cohort of CMML patients (cBioPortal) regarding clinical, epidemiologic, and hematologic features of *KRAS*-mutated patients in order to obtain information on the consistency and general validity of findings.

Oncogenic mutations in the *RAS* genes are present in approximately 30% of all human cancers [17]. Mutations in codons 12, 13, or 61 of one of the three *RAS* genes—*HRAS, KRAS,* and *NRAS*—convert these genes into active oncogenes [18]. The highest incidences are found in adenocarcinomas of the pancreas (90%), colon (50%), and lung (30%); in thyroid tumors (50%); and in myeloid leukemia (30%) [18]. For unexplained reasons, *KRAS* mutations are predominantly found in pancreatic cancer, colorectal cancer, and adenocarcinoma of the lung, whereas mutated *NRAS* is predominantly found in a subset of acute leukemias and in myelodysplastic syndromes [19]. In our study, the percentages of *KRAS* mutations were 9.7% in the ABCMML group and 14.0% in the BIOPOPRTAL group. In the ABCMML cohort, patients with CMML-associated AML had, with 21.7%, an even higher percentage.

Although *NRAS* and *KRAS* share a similar signaling pathway, the impact on clinical outcome and phenotype in CMML is quite different. Whereas the *NRAS* mutation is an established inverse prognostic factor in CMML, in both of our cohorts, the presence of *KRAS* mutations was not associated with an inferior outcome. Moreover, the presence of *KRAS* mutations was not associated with leukocytosis > 13 G/L, whereas patients with *NRAS* mutations have higher leukocyte values than nonmutated patients [13]. Differential effects of oncogenic *KRAS *and *NRAS* mutations on proliferation, differentiation, and tumor progression in the colon have been reported in genetically engineered mice [20]. Expression of *Kras G12D *in the colonic epithelium stimulated hyperproliferation in a Mek-dependent manner, whereas *Nras G12D* did not alter the growth properties of the epithelium but was able to confer resistance to apoptosis. These data indicate that the effects of *KRAS* and *NRAS* may differ in a tissue-specific context.

In a previously reported study, we investigated three cohorts of 337 CMML patients: patients without (A) and with progression/transformation during follow-up (B) and patients already transformed at the time of sampling (C) [20]. The frequencies of *KRAS* mutations in cohorts A, B, and C were 6.1%, 5.3%, and 19% and of *NRAS* mutations 9.1%, 22.8%, and 25.9%, respectively [21]. These data indicate that *KRAS* mutations may play a pathophysiologic role in the course of CMML and its transformation later than *NRAS*.

One limitation of this study is the fact that the proportion of patients with leukocytes > 13 G/L was significantly higher in the ABCMML cohort as compared to the cBioPortal cohort. The reason for this imbalance is not completely clear. Increased laboratory screening in asymptomatic persons in recent years may detect some diseases, including CMML, in an earlier phase than in the past. Therefore, older patient series may be enriched in patients with more advanced disease as compared to more recent series. In fact, we have seen a significant drop in the proportion of patients with MP-CMML from 66% to 48% since 2010 in the ABCMML cohort (unpublished data). Regarding differences in the median survival of patients in the ABCMML and cBioPortal cohorts, we demonstrated in a retrospective study that in patients with CMML, the median overall survival increased from 10 months before 2000 to 23 months thereafter [22]. In this study, AZA-treated patients had improved survival as compared to CMML patients without AZA therapy. Therefore, the fact that in the Austrian database many patients were included in the pre-hypomethylating agent era may explain the differences in median survival between the ABCMML and cBioPortal cohorts.

Changes over time in the diagnostic criteria of CMML since its first description in 1982 represent another limitation of the ABCMML database, suggesting that this patient group is more heterogenous as compared to the cBioPortal group which contains patients who were included over a shorter period of time. Furthermore, it needs to be considered that a proportion of patients in ABCMML, in particular older patients, did not consent to bone marrow (BM) puncture. However, we do not think that this greatly affected diagnostic accuracy, since persistent peripheral blood monocytosis is the most important diagnostic feature, and a genoclinical model has recently been described that uses mutational data, peripheral blood values, and clinical variables to predict the MDS vs. CMML diagnosis with high accuracy in the absence of a BM biopsy result [22]. Moreover, somatic mutations associated with CMML were not only detected in CMML patients confirmed by BM biopsy but also in 57% of patients with nondiagnostic BM features. Interestingly, the OS in mutated patients not confirmed by BM biopsy was indistinguishable from that of patients confirmed by BM biopsy, suggesting that the mutational spectrum is a much more sensitive parameter for detection of myeloid malignancies than BM morphology [23].

In our study, we could confirm the dismal prognosis of CMML-associated AML [24]. The median OS was 5 months in patients without *KRAS *mutations and only 2 months in patients with *KRAS *mutations, although this difference was not statistically different (*p* = 0.655). Thus, it is extremely important to identify patients with a high risk of transformation. In the CPSS-Mol model, using molecular markers such as *RUNX1, NRAS, SETBP1*, and *ASXL1* in addition to clinical and cytogenetic parameters identified four risk groups with a cumulative incidence of leukemic evolution of 0%–48% at 4 years [25]. In patients with high-risk disease, allogeneic hematopoietic cell transplantation (allo-HCT) should be considered because it remains the only potentially curative option. The inherent toxicity of this procedure, however, makes the decision to proceed to allo-HCT challenging, particularly because patients with CMML are mostly older and comorbid. Recently, the European Society for Blood and Marrow Transplantation (EBMT) Practice Harmonization and Guidelines (PH&G) Committee provided the first best-practice recommendations regarding the role of allo-HCT specifically in CMML based on the results of an international survey [26]. For patients unfit for this treatment, hypomethylating agents in combination with targeted treatment will become more and more important in patients with druggable molecular features [27].

## Supplementary Information

Suppl Table 1: Characteristics of patients with CMML without transformation

Suppl Table 2: *KRAS* variants and variant allele frequencies in CMML patients without transformation

Suppl Table 3: Characteristics in patients with CMML-associated AML from the ABCMML database

Suppl Table 4: *KRAS* variants and variant allele frequencies in patients with CMML-associated AML from the ABCMML
