# Supplementary material for: Features of KRAS-mutated patients with chronic myelomonocytic leukemia with and without blast transformation in a national (ABCMML) and international cohort (BIOPORTAL)
Source: Wien Med Wochenschr. 2025 Jul 22;175(11-12):274–81. doi: 10.1007/s10354-025-01099-3 (PMC12380988; doi:10.1007/s10354-025-01099-3)
Supplement: Supplementary file 1 — Suppl Table 1: Characteristics of patients with CMML without transformation [file 10354_2025_1099_MOESM1_ESM.docx]

**Suppl Table 1**: Characteristics of patients with CMML without transformation

| CMML-ABCMML cohort | | |
| --- | --- | --- |
|  | Cases  N=327 | Percent |
| Age  Evaluable = 327 |  |  |
| <70 years | 114 | 35% |
| >70 years | 213 | 65% |
| Sex  Evaluable = 327 |  |  |
| Male | 204 | 62% |
| Female | 123 | 38% |
| Leukocytes  Evaluable = 316 |  |  |
| >13 G/L | 152 | 48% |
| <13 G/L | 164 | 52% |
| Hemoglobin  Evaluable = 316 |  |  |
| <10 g/dL | 100 | 32% |
| >10 g/dL | 216 | 68% |
| Platelets  Evaluable = 317 |  |  |
| <100 G/L | 136 | 43% |
| >100 G/L | 181 | 57% |
| PB Blasts  Evaluable = 265 |  |  |
| absent | 204 | 77% |
| present | 61 | 23% |
| CMML-BIOPORTAL cohort | | |
|  | Cases  N=399 | Percent |
| Age  Evaluable = 399 |  |  |
| <70 years | 136 | 34% |
| >70 years | 263 | 66% |
| Sex  Evaluable = 399 |  |  |
| Male | 268 | 67% |
| Female | 130 | 33% |
| Leukocytes  Evaluable = 383 |  |  |
| >13 G/L | 121 | 32% |
| <13 G/L | 262 | 68% |
| Hemoglobin  Evaluable = 397 |  |  |
| <10 g/dL | 147 | 37% |
| >10 g/dL | 250 | 63% |
| Platelets  Evaluable = 392 |  |  |
| <100 G/L | 155 | 40% |
| >100 G/L | 237 | 60% |
| PB Blasts  Evaluable = 333 |  |  |
| absent | 245 | 74% |
| present | 88 | 26% |
|  |  |  |
