# Supplementary material for: Features of KRAS-mutated patients with chronic myelomonocytic leukemia with and without blast transformation in a national (ABCMML) and international cohort (BIOPORTAL)
Source: Wien Med Wochenschr. 2025 Jul 22;175(11-12):274–81. doi: 10.1007/s10354-025-01099-3 (PMC12380988; doi:10.1007/s10354-025-01099-3)
Supplement: Supplementary file 2 — Suppl Table 2: KRAS variants and variant allele frequencies in CMML patients without transformation [file 10354_2025_1099_MOESM2_ESM.docx]

**Suppl Table 2:** *KRAS* variants and variant allele frequencies in CMML patients without transformation

| **ABCMML ID** | **KRAS** | **VAF** |
| --- | --- | --- |
| CMML_1_086 | A146T | 42 |
| CMML_1_095 | G12D | 45 |
| CMML_1_120 | A12D | 13 |
| CMML_1_133 | G12R | 25 |
| CMML_1_147 | G13D | 26 |
| CMML_1_148 | G12D | 45 |
| CMML_14_302 | Q22K | 13 |
| CMML_17_358 | A18D | 48 |
| CMML_16_361 | G12S | 7 |
| CMML_18_431 | G12V | 52 |
| CMML_18_524 | L15F | 42 |
| CMML_1_528 | T58I | 28 |
| CMML_14_558 | G12C | 37 |
| CMML_1_565 | G12S | 48 |
| CMML_1_596 | G13C | 5 |
| CMML_14_635 | G12R | 42 |
| CMML_3_645 | A146T | 12 |
| CMML_1_650 | G12R | 25 |
| CMML_1_652 | G12A | 24 |
| CMML_18_668 | G12V | 20 |
| CMML_18_669 | G12D | 40 |
| CMML_18_672 | G12S | 7 |
| CMML_18_676 | T58I | 28 |
| CMML_1_695 | G13N | 39 |
| CMML_1_707 | A145P | 9 |
| CMML_16_329 | T58I | 14 |
| CMML_17_350 | G60V | 48 |
| CMML_17_353 | D33E | 9 |
| CMML_1_463 | Q22K | 7 |
| CMML_1_470 | Q61R | 41 |
| CMML_1_538 | G12D | 7 |
| CMML_4_619 | A18N | 38 |

| **BIOPORTAL ID** | **KRAS** | **VAF** |
| --- | --- | --- |
| E-H-100086 | G12A | 4 |
| E-H-100137 | Q61R | 6 |
| E-H-100187 | G12R | 46 |
| E-H-100194 | G12R | 28 |
| E-H-100197 | K117N | 7 |
| E-H-100206 | G12D | 24 |
| E-H-100220 | G12D | 15 |
| E-H-100317 | K117N | 39 |
| E-H-100361 | G60V | 8 |
| E-H-100393 | T58I | 48 |
| E-H-100426 | G13D | 30 |
| E-H-102606 | Q61P | 4 |
| E-H-102619 | G12R | 41 |
| E-H-103070 | L19F | 20 |
| E-H-103082 | G12A | 45 |
| E-H-105577 | G12S | 47 |
| E-H-105724 | T58I | 31 |
| E-H-105764 | A146V | 21 |
| E-H-106240 | A146T | 7 |
| E-H-110410 | K117N | 2 |
| E-H-110416 | G12S | 28 |
| E-H-110719 | G12D | 14 |
| E-H-110759 | Q61H | 33 |
| E-H-110785 | A18D | 3 |
| E-H-110787 | G12D | 43 |
| E-H-110811 | G12D | 44 |
| E-H-110824 | L19F | 31 |
| E-H-110840 | A146P | 2 |
| E-H-110923 | A146T | 23 |
| E-H-116356 | G13D | 46 |
| E-H-116397 | G12D | 38 |
| E-H-116434 | G12R | 35 |
| E-H-116443 | G12R | 16 |
| E-H-116450 | G12R | 38 |
| E-H-116521 | T58I | 3 |
| E-H-116552 | G12A | 27 |
| E-H-116678 | K117N | 45 |
| E-H-116743 | G12R | 6 |
| E-H-116768 | G60D | 7 |
| E-H-116801 | K117N | 39 |
| E-H-117990 | D33E | 4 |
| E-H-118011 | G13D | 30 |
| E-H-118026 | K117N | 27 |
| E-H-118064 | Q61R | 28 |
| E-H-118204 | A146T | 9 |
| E-H-118517 | G12D | 42 |
| E-H-118777 | Q61L | 5 |
| E-H-118782 | A146P | 5 |
| E-H-120815 | G12S | 46 |
| E-H-121080 | G12S | 4 |
| E-H-121090 | G12R | 45 |
| E-H-121115 | A146T | 41 |
| E-H-121147 | G12D | 16 |
| E-H-131504 | G12R | 38 |
| E-H-131511 | K117N | 41 |
