# Supplementary material for: Features of KRAS-mutated patients with chronic myelomonocytic leukemia with and without blast transformation in a national (ABCMML) and international cohort (BIOPORTAL)
Source: Wien Med Wochenschr. 2025 Jul 22;175(11-12):274–81. doi: 10.1007/s10354-025-01099-3 (PMC12380988; doi:10.1007/s10354-025-01099-3)
Supplement: Supplementary file 3 — Suppl Table 3: Characteristics in patients with CMML-associated AML from the ABCMML database [file 10354_2025_1099_MOESM3_ESM.docx]

**Suppl Table 3**: Characteristics in patients with CMML associated AML from the ABCMML database

|  | Cases  N=46 | Percent |
| --- | --- | --- |
| Age  Evaluable = 46 |  |  |
| <70 years | 18 | 39% |
| >70 years | 28 | 61% |
| Sex  Evaluable = 46 |  |  |
| Male | 27 | 59% |
| Female | 19 | 41% |
| Leukocytes  Evaluable = 41 |  |  |
| >13 G/L | 34 | 83% |
| <13 G/L | 7 | 17% |
| Hemoglobin  Evaluable = 41 |  |  |
| <10 g/dL | 22 | 54% |
| >10 g/dL | 19 | 46% |
| Platelets  Evaluable = 41 |  |  |
| <100 G/L | 31 | 76% |
| >100 G/L | 10 | 24% |
| PB Blasts  Evaluable = 40 |  |  |
| absent | 11 | 28% |
| present | 29 | 72% |
