# Supplementary material for: Features of KRAS-mutated patients with chronic myelomonocytic leukemia with and without blast transformation in a national (ABCMML) and international cohort (BIOPORTAL)
Source: Wien Med Wochenschr. 2025 Jul 22;175(11-12):274–81. doi: 10.1007/s10354-025-01099-3 (PMC12380988; doi:10.1007/s10354-025-01099-3)
Supplement: Supplementary file 4 — Suppl Table 4: KRAS variants and variant allele frequencies in patients with CMML-associated AML from the ABCMML [file 10354_2025_1099_MOESM4_ESM.docx]

**Suppl Table 4:** *KRAS* variants and variant allele frequencies in patients with CMML associated AML from the ABCMML

| **ABCMML ID** | **KRAS** | **VAF** |
| --- | --- | --- |
| CMML_16_334_AML | A18D | 48 |
| CMML_16_335_AML | G12S | 48 |
| CMML_18_422_AML | G12S | 22 |
| CMML_1_044_AML | T58I | 37 |
| CMML_16_371_AML | G12D | 41 |
| CMML_1_534_AML | G12A | 35 |
| CMML_4_613_AML | Q61H | 40 |
| CMML_3_647_AML | NA | 46 |
| CMML_1_686_AML | G13D | 14 |
| CMML_14_300_AML | Q61R | 9 |
